# 

**Published:** 1814-07

**Authors:** 


### MEDICAL AND PHYSICAL JOURNAL.

CONDUCTED BY

SAMUEL FOTHERGILL, M.D.
PHYSICIAN EXTRAORDINARY TO HIS ROYAL HIGHNESS THE DUKE OF KENT;
PHYSICIAN TO THE ASYLUM FOR FEMALE ORPHANS; AND TO THE
WESTMINSTER GENERAL, AND THE WESTERN, DISPENSARIES.

VOL. XXXII.

FROM JULY TO DECEMBER, 1814.

Et quoniam variant morbi, variabimus artes;
Mille mali species, mille salutis erunt.

5138

*LONDON:*

PRINTED FOR THE PROPRIETORS, BY J. ADLARD, 23, BARTHOLOMEW-CLOSE,
AND 39, DUKE-STREET, SMITHFIELD;
AND PUBLISHED BY J. SOUTER, NO. 1, PATERNOSTER-ROW.

1815.

### MONTHLY CATALOGUE OF MEDICAL BOOKS.

ELEMENTS of Medical Jurisprudence; or, a succinct and compendious Description of such Tokens in the Human Body as are requisite to determine the Judgment of a Coroner, and of Courts of Law, in Cases of Divorce, Rape, Murder, &c. To which are added, Directions for preserving the Public Health. By Samuel Farre, M.D. Second Edition. 12mo. boards. 5s.

An Essay on Bronchitis; with a Supplement: containing Remarks on Simple Pulmonary Abscess, &c. &c. By Charles Badham, M.D. Physician of the Household to his Royal Highness the Duke of Sussex, formerly Physician to the Westminster General Dispensary, &c. &c. Second Edition, corrected and enlarged. 12mo. boards. 5s.

### TO CORRESPONDENTS.

*We are sorry that Dr. H.'s Letter came too late to be attended to.*

